# Current Assessment of the Effects of Environmental Chemicals on the Mammary Gland in Guideline Rodent Studies by the U.S. Environmental Protection Agency (U.S. EPA), Organisation for Economic Co-operation and Development (OECD), and National Toxicology Program (NTP)

**DOI:** 10.1289/ehp.1002676

**Published:** 2010-11-30

**Authors:** Susan L. Makris

**Affiliations:** National Center for Environmental Assessment, Office of Research and Development, U.S. Environmental Protection Agency, Washington, DC, USA

**Keywords:** carcinogenicity study, endocrine-disrupting chemicals, extended one-generation reproduction study, mammary gland, perinatal exposure, risk assessment, subchronic toxicity study, toxicity testing guidelines, two-generation reproduction study

## Abstract

Background: Evaluation of the structural and/or functional integrity of the mammary gland (MG) across life stages is integral to the assessment of developmental, reproductive, and carcinogenic risk for environmental chemicals.

Objectives: In this commentary I characterize MG assessment recommended in U.S. Environmental Protection Agency, Organisation for Economic Co-operation and Development, and National Toxicology Program guideline toxicology study protocols and identify any information gaps for the evaluation of MG development, structure, and function.

Discussion: Several data gaps, issues, and challenges were identified. Current guidelines that include a lactation phase do not provide specific recommendations to record observations on maternal or offspring lactation or nursing behavior. In guideline studies, the assessment of MG toxicity often relies upon indirect, nonspecific, or surrogate end points, and information that could be useful in the interpretation of these data (e.g., mode of action or toxicokinetics) is often unavailable. Most guideline studies designed to assess general organ toxicity do not expose test animals during sensitive stages of MG development; histopathological evaluation of the developing MG is not routinely conducted; and evaluation of MG tissue for both sexes is inconsistently recommended.

Conclusions: I propose the following general recommendations to enhance MG assessment in guideline toxicology studies: *a*) inclusion of more specific criteria for the evaluation of MG end points in guideline language, *b*) inclusion of histopathological evaluation of MG development (using whole-mount techniques) in existing or new guideline protocols that include offspring with perinatal and/or pubertal treatment, *c*) incorporation of perinatal exposures into rodent subchronic and carcinogenicity assays, and *d*) expansion of the histopathological evaluation of male MG tissue.

Evaluation of the structural and functional integrity of the mammary gland (MG), an important reproductive tissue/organ, is integral to the assessment of developmental, reproductive, and carcinogenic risk for environmental chemicals [Organisation for Economic Co-operation and Development (OECD) 2008; U.S. Environmental Protection Agency (EPA) 1991, 1996, 2005a, 2005b]. When using a life-stage–oriented approach to risk assessment, it is important to assess early developmental periods (including *in utero* through puberty), function during reproductive life stages, and continued health in later life stages.

The evidence for alterations in MG development, lactational function, and cancer risk is reviewed by Rudel et al. (2011). Examples of hormonally active agents that are associated with MG alterations include diethylstilbestrol (DES), genistein, atrazine, bisphenol A (BPA), dibutyl phthalate, dioxin, methoxychlor, nonylphenol, polybrominated diphenyl ethers (PBDEs), and perfluorooctanoic acid (PFOA).

Animal studies are traditionally used in predicting potential toxicity and risk to humans. The use of this approach to screen environmental chemicals for MG toxicity is supported by parallels between MG effects induced in animal models and alterations in human MG health. Examples include altered timing of puberty, alterations in lactation (e.g., ability to lactate, quality and quantity of milk), and induction of mammary/breast cancer.

Standardized study designs or protocols (i.e., guidelines) are often used to screen chemicals for adverse effects related to chemical exposures. Examples are those published by the U.S. EPA, OECD, and National Toxicology Program (NTP). The application and use of these guideline studies generally depend on the regulatory agency and its applicable legislative mandates for specified chemical classes and their anticipated uses or potential human exposures. The arsenal of study types is relatively consistent across organizations and programs, partly because testing paradigms are established on accepted biological principles and validated experimental procedures, and partly because of concerted efforts to develop and maintain consistency (e.g., through the guideline harmonization program implemented by the U.S. EPA and OECD). Some guidelines include assessment of MG end points. Here I characterize MG assessment in guideline study protocols used in chemical testing and identify any information gaps in the testing paradigm.

## Materials and Methods

For this evaluation, I selected published U.S. EPA, OECD, and NTP toxicity testing guidelines in rodents. These guidelines were developed with a rigorous peer-review process and have a long-standing history of use, interpretation, and application in data evaluation and risk assessment. U.S. EPA and OECD guidelines, although “harmonized,” were both included here because there are some minor but important differences.

I used guidelines available on the U.S. EPA, OECD, and NTP web sites (NTP 2008; OECD 2011; U.S. EPA 2010b) as source documents for the analysis of MG testing recommendations. Because MG is a reproductive tissue, guideline studies designed to provide some assessment of reproductive system structure or function (including developmental outcomes) have been identified (Table 1). They fall into several general categories: studies that *a*) include an assessment of reproductive function and outcome, *b*) evaluate endocrine-related effects, and *c*) examine general organ toxicity and pathology after less-than-lifetime (subacute or subchronic) or after long-term (chronic or lifetime) chemical exposures.

**Table 1:** U.S. EPA, OECD, and NTP guidelines that assess developmental and reproductive toxicity in mammalian species.

Guideline	U.S. EPA	OECD	NTP
Studies that include assessments of reproductive function and outcome						
Prenatal developmental toxicity		OPPTS 870.3700 (U.S. EPA 1998e)		TG 414 (OECD 2001a)		*a*
One-generation reproduction		*b*^,c^		TG 415 (OECD 1983)		*b*
Reproduction and fertility effects (two-generation reproduction)		OPPTS 870.3800 (U.S. EPA 1998f)		TG 416 (OECD 2001b)		*b*
RACB		*b*		*b*		*a*
Reproduction/developmental toxicity screening test		OPPTS 870.3550 (U.S. EPA 2000a)		TG 421 (OECD 1995)		*b*
Combined repeated dose toxicity study with the reproduction/developmental toxicity screening test		OPPTS 870.3650 (U.S. EPA 2000b)		TG 422 (OECD 1996)		*b*
DNT		OPPTS 870.6300 (U.S. EPA 1998j)		TG 426 (OECD 2007a)		*b*
Endocrine screening and testing studies						
Uterotrophic assay*d*		OPPTS 890.1600 (U.S. EPA 2009d)		TG 440 (OECD 2007b)		*b*
Hershberger assay*d*		OPPTS 890.1400 (U.S. EPA 2009a)		TG 441 (OECD 2009c)		*b*
Juvenile/peripubertal male assay*d*		OPPTS 890.1500*b*^,c ^(U.S. EPA 2009c)		*b*		
Juvenile/peripubertal female assay*d*		OPPTS 890.1450*b*^,c ^(U.S. EPA 2009b)		*b*		
General organ toxicity assessments after less-than-lifetime exposures						
14-Day oral toxicity in rodents*e*		*b*		*b*		*a*
28-Day oral toxicity in rodents*e*		OPPTS 870.3050 (U.S. EPA 2000a)		TG 407 (OECD 2005)		*b*
21/28-Day dermal toxicity*e*		*b*		TG 410 (OECD 1981a)		*b*
28-Day inhalation toxicity*e*		*b*		TG 412 (OECD 2009a)		*b*
90-Day oral toxicity in rodents*e*		OPPTS 870.3100 (U.S. EPA 1998a)		TG 408 (OECD 1998a)		*a*
90-Day oral toxicity in nonrodents*e*		OPPTS 870.3150 (U.S. EPA 1998b)		TG 409 (OECD 1998b)		*b*
90-Day dermal toxicity*e*		OPPTS 870.3250 (U.S. EPA 1998c)		TG 411 (OECD 1981b)		*b*
90-Day inhalation toxicity*e*		OPPTS 870.3465 (U.S. EPA 1998d)		TG 413 (OECD 2009b)		*b*
General organ toxicity assessments after long-term or lifetime exposures						
Chronic toxicity*e*		OPPTS 870.4100 (U.S. EPA 1998g)		TG 452 (OECD 2009e)		*b*
Carcinogenicity*e*		OPPTS 870.4200 (U.S. EPA 1998h)		GL 451 (OECD 2009d)		*b*
Combined chronic toxicity/carcinogenicity*e*		OPPTS 870.4300 (U.S. EPA 1998i)		GL 453 (OECD 2009f)		*a*
Combined chronic toxicity/carcinogenicity testing of respirable fibrous particles*e*		OPPTS 870.8355 (U.S. EPA 2001)		*b*		*b*
Abbreviations: OPPTS, Office of Prevention, Pesticides and Toxic Substances; TG, Test Guideline. **a**Based on NTP guidelines available online (NTP 2008); the NTP does not catalog or identify guidelines using a numerical coding system. **b**No comparable assay listed on agency/organization web site. **c**U.S. EPA and OECD collaborative effort on guideline development. **d**Endocrine screening assay. **e**Although not traditionally considered to be a reproductive toxicity assay, the protocol contains reproductive system end points that should be considered in a weight-of-evidence evaluation of developmental and reproductive toxicity.						

The types of MG assessments in these guideline studies include direct measurements of MG structure or function, as well as measurements of biomarkers that provide surrogate indicators of potential effects on the MG (Table 2). Each guideline protocol was examined to ascertain which MG assessments are explicitly or implicitly recommended.

**Table 2:** Evaluation of MG structure and function in rodent toxicology studies.

Direct structural or functional measurements
Clinical observations, palpation*a*
Macroscopic pathology at necropsy*a*
Microscopic pathology*a*
Lactation assessments
Maternal breast enlargement
Maternal and pup nursing behavior
Pup survival and body weight gain*b*
Presence of “milk spot” in pups
Measurement of milk composition
Measurements of related biomarkers
Endocrine-sensitive developmental landmarks
Anogenital distance
Nipple retention in males
Preputial separation
Vaginal patency
Endocrine-sensitive reproductive evaluation
Estrous cyclicity
**a**Adult assessments. **b**Pup weight gain and survival can be surrogate indicators of normal lactation.

## Results

*Studies including assessments of reproductive function and outcome.* U.S. EPA, OECD, and NTP rodent protocols that include an assessment of reproductive function and an examination of the production and maintenance of offspring also include a lactation phase and thus provide some degree of MG assessment. Guidelines that include a lactation phase are the reproduction/developmental toxicity screening test (with and without a repeated dose toxicity segment) (OECD 1995, 1996; U.S. EPA 2000b, 2000c), the one-generation reproduction study (OECD 1983), the two-generation reproduction study (OECD 2001b; U.S. EPA 1998f), the developmental neurotoxicity (DNT) study (OECD 2007a; U.S. EPA 1998j), and the NTP reproductive assessment by continuous breeding (RACB) (Chapin and Sloane 1996). I also considered the draft OECD extended one-generation reproduction study (OECD 2010) in this analysis because the protocol has been developed as a collaborative harmonized effort between the OECD and the U.S. EPA, and it is currently undergoing public and peer-review processes expected to culminate in finalization. I did not include the prenatal developmental toxicity study guideline in this list because it specifies termination of the dam and fetuses before expected delivery and does not include a lactation phase or direct assessment of MG function in dams or postnatal development in offspring.

Brief descriptions of the selected protocols follow. The U.S. EPA and OECD protocols are illustrated in Supplemental Material, Figure 1 (doi:10.1289/ehp.1002676).

- The U.S. EPA/OECD reproduction/developmental screening studies are intended to generate initial toxicity information and prioritize the need for further testing of industrial chemicals; they are also used in toxicological screening of pesticide inert ingredients. These screening protocols include a single mating. Pups are maintained only to postnatal day (PND) 4, at which time the study is terminated; end point assessments are limited in number and scope.
- The OECD one-generation reproduction study includes a single mating; however, the litters are maintained and evaluated until the time of weaning (PND21). This protocol, which is sometimes used as a preliminary screening or dose range–finding study, does not include endocrine end points that were added to the U.S. EPA and OECD two-generation reproduction study guidelines during updating and harmonization efforts conducted in the late 1990s.
- The U.S. EPA/OECD DNT study includes a single mating period, and offspring are maintained throughout the lactation period and kept on study until approximately PND70. The primary focus of this study is the assessment of neurobehavioral development and neuropathology of the offspring, rather than on the reproductive system, and observations of the maternal animals are not extensive.
- In the U.S. EPA/OECD two-generation reproduction study (also called a reproduction and fertility effects study), weanling offspring from the first generation are selected as parental animals for the second generation. Thus, data from two lactation periods are typically available. This study includes enhanced reproductive, endocrine, and postmortem data that originated from guideline harmonization efforts.
- The draft extended one-generation reproduction study under collaborative development by OECD and U.S. EPA is intended to provide an enhanced alternative method for developmental/reproductive toxicity screening that incorporates DNT and developmental immunotoxicity testing, assesses endocrine end points, and enhances postmortem evaluations, while at the same time using fewer animals and using them more efficiently than traditional guideline studies.
- The NTP RACB study is an enhancement to a two-generation reproduction study protocol that is designed around the conduct of targeted study segments or “tasks.” Task 1 is a dose range–finding study; task 2 is a continuous 14-week breeding segment using serial matings to determine fertility and reproductive outcome, with early postnatal termination of the first sets of litters and then maintenance of the final litter to the age of weaning (Chapin and Sloane 1996); task 3 incorporates the use of naive females or control males in crossover matings with high-dose animals to assess possible sex- and treatment-related reproductive effects; and task 4 initiates the production of a second generation.

Overall, none of these study protocols focus on end points specific to MG development, health, or successful nursing behaviors. However, they all include recommendations for regular scheduled clinical observations of both parental animals and offspring, including during periods of lactation. In a well-conducted toxicology study, such clinical observations should be able to detect gross (but perhaps not subtle) abnormalities in maternal mammary tissue conformity and function, as well as disruptions in normal nesting and nursing behaviors. Many of the studies also include necropsy of the parental animals, which might be expected to identify macroscopic abnormalities of the mammary tissue; histopathological evaluation of abnormal reproductive system tissues is not specified for the reproduction/developmental toxicity screening test or for the DNT study. Compromised health status of pups may also provide some indication of physical or behavioral alterations to maternal lactation. For example, delayed growth (body weight) in pups, an absence of evidence of milk in the stomach of very young rodent pups (which can be visualized externally as a “milk spot” or “milk band”), or offspring dehydration and morbidity may be indicative of malnutrition or interrupted nursing behavior.  There is generally insufficient information to determine if the effect is due to physiological or behavioral alterations in maternal lactation or direct toxicity to the pups that compromises their ability to nurse or thrive.

Most of the protocols in this category include some assessment of endocrine- mediated developmental or reproductive biomarkers that can be informative regarding disruptions in MG development or function (exceptions being the U.S. EPA/OECD developmental/reproduction screening tests and the OECD one-generation reproduction study). The age of offspring sexual maturation is recorded in the U.S. EPA/OECD DNT study, and estrous cyclicity is evaluated in the NTP RACB study. A number of endocrine-mediated end points are assessed in the two-generation reproduction study: age of sexual maturation, anogenital distance measurements (when triggered by other adverse findings), and estrous cyclicity. The draft OECD extended one-generation reproduction study expands upon this list by including an assessment of the age at sexual maturation, anogenital distance measurements (in all offspring; i.e., not triggered by other adverse findings as in the two-generation reproduction study), evaluations of nipple/areola retention in male pups, and estrous cyclicity.

*Studies used in endocrine screening and testing.* Endocrine screening programs for environmental toxicants, using a tiered testing approach, have been initiated by the U.S. EPA and OECD. Tier 1 tests consist of a battery of assays designed to efficiently and effectively screen chemicals for interactions with the estrogen, androgen, or thyroid hormonal systems. If a weight-of-evidence evaluation of the results from the Tier 1 assays indicates potential interaction with these hormonal systems, then additional, more comprehensive screening would be implemented in Tier 2 testing. Assays that have been validated for use in Tiers 1 and 2 are listed in Table 3. The overall endocrine profile for a chemical may provide important information regarding the potential for MG toxicity or disruption of MG development. However, the evaluation of MG tissue or function is seldom addressed in the endocrine screening protocols. A review of the Tier 1 guidelines identified only four *in vivo* mammalian studies, none of which included a lactation phase with potential assessment of MG function [see Supplemental Material, Figure 2 (doi:10.1289/ehp.1002676)]. The uterotrophic assay (OECD 2007b; U.S. EPA 2009d) is designed to screen for (anti)estrogenic activity in ovariectomized or immature female rats; the Hershberger assay (OECD 2009c; U.S. EPA 2009a) screens for androgenic activity in castrated peripubertal male rats; and the male and female pubertal assays (U.S. EPA 2009b, 2009c) evaluate (anti)androgenic plus thyroid activity in male rats or estrogenic plus thyroid activity in female rats during sexual maturation. Although these studies include general clinical and necropsy observations, the evaluations do not focus on palpation or examination of mammary tissue, and histopathology of abnormal mammary tissue or of mammary tissue from animals treated during MG development is not specified. Notably, optional assessments of serum levels of reproductive hormones are included in the Hershberger and pubertal assays; these data are possible biomarkers of endocrine disruption that could be indicative of alterations in MG development, structure, or function. Tier 2 endocrine screening includes the two-generation reproduction study, described above.

**Table 3:** Endocrine disruptor tiered testing approach (U.S. EPA 2010a).

Assay	Species
Tier 1 testing assays		
Amphibian metamorphosis		Frog
Estrogen and androgen receptor binding assays		*In vitro*
Aromatase		*In vitro*
Steroidogenesis		*In vitro*
Fish short-term reproduction screen		Fish
Uterotrophic assay		Rat
Hershberger assay		Rat
Pubertal male assay		Rat
Pubertal female assay		Rat
Tier 2 testing assays		
Amphibian development, reproduction		Frog
Avian two-generation		Japanese quail
Fish life cycle		Fish
Invertebrate life cycle		Mysid shrimp
Mammalian two-generation reproduction		Rat

*Studies examining general organ toxicity and pathology.* Although studies designed to evaluate general organ toxicity and pathology do not typically include a lactation phase [see Supplemental Material, Figure 3 (doi:10.1289/ehp.1002676)], they do include clinical and postmortem assessments of reproductive organs.

Less-than-lifetime (subacute or subchronic) chemical exposures. By definition, subacute studies are approximately 14 or 28 days in duration, and subchronic studies are 3–6 months in duration. These studies are typically used to establish dose levels for subsequent longer-term studies or to identify target organ toxicity. There are NTP guidelines for 14- and 90-day studies (NTP 2010b) and U.S. EPA and OECD guidelines for 28- and 90-day studies (OECD 1998a, 2005; U.S. EPA 1998a, 2000a). These studies are generally conducted in young adult animals. Abnormalities in mammary tissues may be detected in either sex by palpation during in-life clinical observations (conducted at least weekly) or may be observed at necropsy. Study protocols provide lists of organs/tissues for dissection, fixation, sectioning, and microscopic examination and specify histopathological evaluation of abnormal tissues. MG evaluation is not consistently recommended across U.S. EPA, OECD, and NTP subacute and subchronic guidelines. The NTP 14-day guideline recommends histopathology of abnormal MG tissue for either sex. The U.S. EPA 28-day guideline (U.S. EPA 2000a) does not mention MG assessment. However, the OECD 28-day guideline (OECD 2005) includes a list of tissues intended to provide indicators for endocrine-related effects, specifying evaluation of male (but not female) MG tissue, and it states that “changes in male mammary glands have not been sufficiently documented but this parameter may be very sensitive to substances with estrogenic action.” In the 90-day subchronic guidelines, the U.S. EPA and OECD recommend assessment only of female mammary tissue, whereas the NTP recommends evaluation of abnormal tissue in both sexes.

Long-term (chronic) or lifetime exposures. Long-term studies in rodents are > 6 months in duration. The duration of studies that are designed to approximate lifetime exposure to a chemical and that focus on assessment of carcinogenicity is at least 18 months in the mouse and 24 months in the rat. In addition to regular (at least weekly) clinical observations in these studies, macroscopic observations are collected at interim and terminal sacrifice, and histopathology of MG tissue is routinely required, whether or not abnormalities are observed in-life or at necropsy. U.S. EPA and OECD protocols (OECD 2009e, 2009f; U.S. EPA 1998g, 1998h, 1998i, 2001) specify MG histopathology only for female rodents, whereas NTP protocols do not make a distinction between sexes (NTP 2010b). It is in long-term studies that MG cancer is usually identified, due partly to *a*) the extended treatment period, *b*) the statistical power of larger group sizes used in these studies (i.e., ≥ 50 rodents/sex/group, compared with ~ 10–20/sex/group in short-term studies), and *c*) the focus on histopathological evaluation, often including rigorous pathology peer review.

Perinatal exposures. Historically, the contribution of early life exposures to toxicity assessments in subacute, subchronic, and chronic studies has not been routinely evaluated for environmental chemicals. Current U.S. EPA and OECD guidelines do not specify that the animals placed on study be exposed to the test chemical during *in utero* or preweaning development; most are placed on study as young adults of 5–6 weeks of age. The NTP, however, has recently taken an important step in this direction by providing detailed information for the application of a perinatal treatment phase in range-finding, 13-week, and 2-year studies in rats (NTP 2010a); other species are not addressed. In this perinatal study design, pregnant dams are exposed to the test substance starting at gestation day 6, and exposure is continued through to weaning of the litters at PND21. Thus, the offspring are exposed to the test substance during postimplantation *in utero* development, during postnatal development via maternal milk, and through direct exposure (i.e., in treated feed or water or by gavage administration). At weaning, selected offspring are assigned to the 13-week and 2-year studies for continuation of treatment.

## Discussion

Table 4 summarizes the extent of MG evaluation in U.S. EPA, OECD, and NTP guideline rodent toxicology studies, focusing on lactation assessments in dams and pups; the evaluation of developmental or reproductive endocrine end points that can serve as biomarkers for MG disruption; and the examination of maternal clinical observation, macroscopic pathology, and histopathology data.

**Table 4:** Summary of MG evaluation in guideline toxicology studies.

Guideline study	Lactation assessment	Endocrine end point	Clinical observation	Macroscopic pathology	Histopathology
Prenatal developmental toxicity						*a*		*a*		
Screening developmental/reproduction		X*b*				*a*		*a*		
Two-generation reproduction		X*b*		X		X		X		X*c*
RACB		X*b*		X		X		X		X*c*
One-generation reproduction		X*b*				X		X		X*c*
Extended one-generation reproduction		X*b*		X		X		X		X
DNT		X*b*		X		*a*		*a*		
Uterotrophic assay						*a*		*a*		
Hershberger assay				X*d*		*a*		*a*		
Juvenile/pubertal male or female assay				X		*a*		*a*		
Subacute or subchronic						X		X		X*c*
Chronic or carcinogenicity						X		X		X
X indicates that the study includes an assessment of mammary end points. **a**Recommended clinical and necropsy evaluations do not focus on palpation or examination of mammary tissue, and subsequent histopathology of abnormal MG tissue is not required. **b**The study includes a lactation phase, but other than the surrogate measurement of pup growth and survival, the guideline does not recommend specific MG end points. **c**Except for OECD Test Guideline 407 (OECD 2005), which includes routine evaluation of male MG as an indicator of endocrine disruption, MG histopathology is not recommended unless triggered by observed gross abnormalities. **d**Assessment of serum hormone levels (thyroxine, triiodothyronine, testosterone, luteinizing hormone, follicle-stimulating hormone) is optional.										

In this analysis, I have identified several data gaps, issues, and challenges:

- Guidelines that include a reproduction phase do not specify that observations on maternal or offspring lactation or nursing behavior should be recorded.
- Many of the functional mammary end points assessed are indirect or nonspecific. For example, observed treatment-related outcomes or indicators of disruption to lactation may be related to or influenced by a variety of confounding factors such as the overall health (or toxicity status) of the maternal animal and/or offspring. Toxicokinetic data on the test substance and/or its metabolite(s) or mode of action data, which might inform this issue, are seldom available.
- When assessments of endocrine function are conducted, they may or may not be indicative of adverse MG outcomes, because this is critically dependent on the mode or mechanism of action of the chemical. This information is unknown for many environmental chemicals.
- Many guideline studies do not evaluate animals that have been exposed during critical periods of MG development. The studies that include such exposures are the reproductive toxicity studies (including the U.S. EPA/OECD one- and two-generation studies and the NTP RACB study), the U.S. EPA peripubertal endocrine assays, the U.S. EPA/OECD DNT study, and the perinatal phase of the NTP rat carcinogenicity assay.
- When the MG is evaluated histopathologically, it is not examined during development; instead, the focus is on adult pathology.
- In short- and long-term studies that evaluate general organ toxicity and pathology, examination of MG tissue is seldom routinely recommended for both sexes; there is generally a preferential focus on evaluation of female MG tissue, even though adverse treatment-related effects could occur in male MG tissue.

To address these issues, a paradigm shift would be needed for the evaluation of MG in guideline studies. Implementing such a shift would present a number of challenges. These include addressing *a*) issues of species and strain sensitivity; *b*) the timing of exposure and assessments; *c*) the sensitivity of end points typically assessed in guideline studies for the detection of effects on MG development or function; and *d*) the statistical power of the study design. Treatment-related effects on MG gland development and/or later life consequences, whether structural or functional and whether transient or permanent, should be considered adverse and relevant to risk assessment; this approach is consistent with U.S. EPA and OECD risk assessment guidelines and practice (OECD 2008; U.S. EPA 1991, 1996).

## Conclusions

This review and analysis of U.S. EPA, OECD, and NTP guidelines for the assessment of environmental toxicants identified the need to expand the focus on MG evaluation in guideline toxicity studies. Several recommendations for enhancing MG evaluation in guideline toxicology studies have been proposed (Rudel et al. 2011).

First, guidelines should be written or revised to more specifically address the examination of MG end points. Second, consideration should be given to including the histopathological evaluation of MG development in existing or new guideline protocols that include offspring treated during *in utero* and postnatal development and that are maintained on study to the age of weaning or puberty. The use of a whole-mount histopathological technique (described by White et al. 2011) is important to this assessment. In some studies, such as the DNT study and the two-generation reproduction study, most offspring are already committed to other assessments. However, two study protocols include a developmental exposure and that are likely to have sufficient dams or pups available for MG whole-mount assessment: the U.S. EPA male and female juvenile/pubertal  assays (where MG could be added to the list of tissues collected and assessed at study termination) and the OECD draft extended one-generation reproduction study [where MG could be assessed in parental generation dams killed after weaning of the F_1_ pups, and in F_1_ offspring at specified time points (e.g., PND4 after litter standardization, PND 21 after weaning and discarding extra pups, and PND90 at study termination)].

Third, early life exposures should be incorporated into U.S. EPA and OECD rodent subchronic and carcinogenicity assays, and the NTP should consider providing guidance on adding perinatal exposure phases to mouse studies, especially because the mouse can be a sensitive model for detecting alterations in MG morphogenesis after *in utero* and/or lactational exposures and for assessing MG carcinogenicity. Finally, consideration should be given to enhancing the histopathological evaluation of male MG tissue, which may be uniquely susceptible to developmental perturbation or risk for breast cancer.

## Supplemental Material

(552 KB) PDF
