# Supplementary material for: Current Assessment of the Effects of Environmental Chemicals on the Mammary Gland in Guideline Rodent Studies by the U.S. Environmental Protection Agency (U.S. EPA), Organisation for Economic Co-operation and Development (OECD), and National Toxicology Program (NTP)
Source: Environ Health Perspect. 2010 Nov 30;119(8):1047–52. doi: 10.1289/ehp.1002676 (PMC3237339; doi:10.1289/ehp.1002676)
Supplement: (552 KB) PDF [file ehp.1002676.s001.pdf]

Supplemental materials for:

Current Assessment of the Effects of Environmental Chemicals on the Mammary Gland in Guideline EPA, OECD, and NTP Rodent Studies

Susan L. Makris

This file contains figures illustrating various study designs:

Figure 1. Studies that include assessments of reproductive function and postnatal outcome

Figure 2. Mammalian *in vivo* endocrine assays

Figure 3. Subacute, subchronic, and chronic/carcinogenicity studies in rodents

Figure 1. Studies that include assessments of reproductive function and postnatal outcome Timelines for study conduct are presented for the reproduction/developmental toxicity screening test (OCED 1995, 1996; U.S. EPA 2000a, b), the one-generation reproduction study (OECD 1983), the two-generation reproduction study (OECD 2001, U.S. EPA 1998a), the developmental neurotoxicity (DNT) study (OECD 2007a, U.S. EPA 1998b), and the draft extended one-generation reproduction study (OECD 2010; source of diagram). In each study except the DNT, treatment is continuous from study initiation through termination. A study illustration for the NTP reproduction assessment by continuous breeding (RACB) study is not presented here, but can be found in Chapin and Sloane, 1996. Key: GD = gestation day; PND = postnatal day; P = parental (first) generation; F1 = first filial (second) generation; M = male; F = female

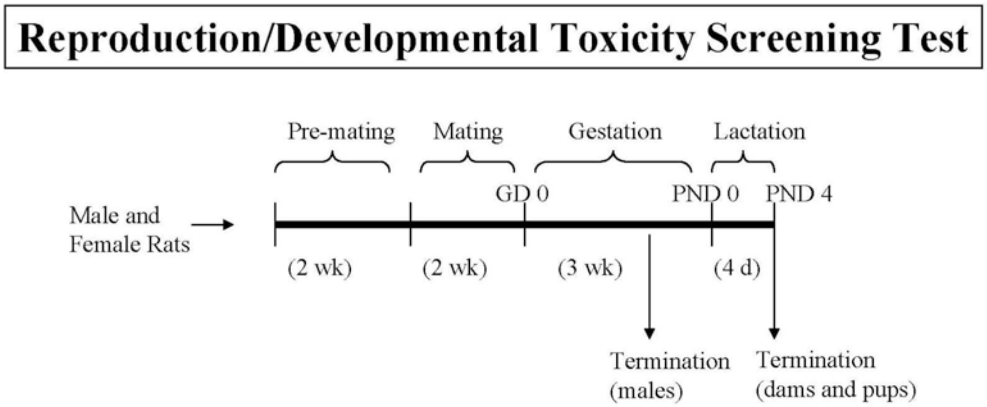

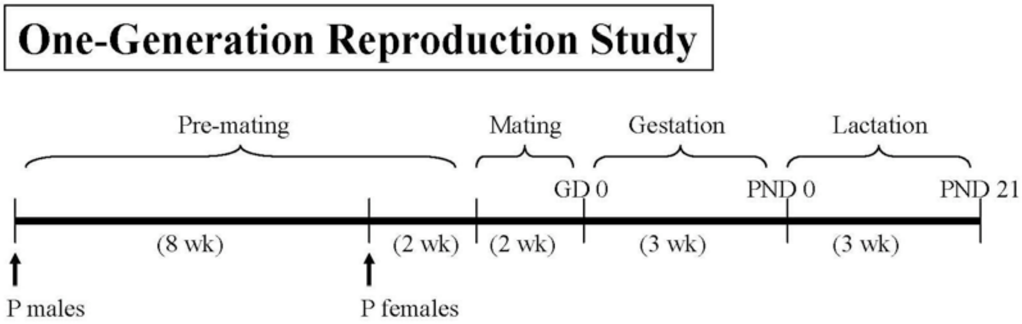

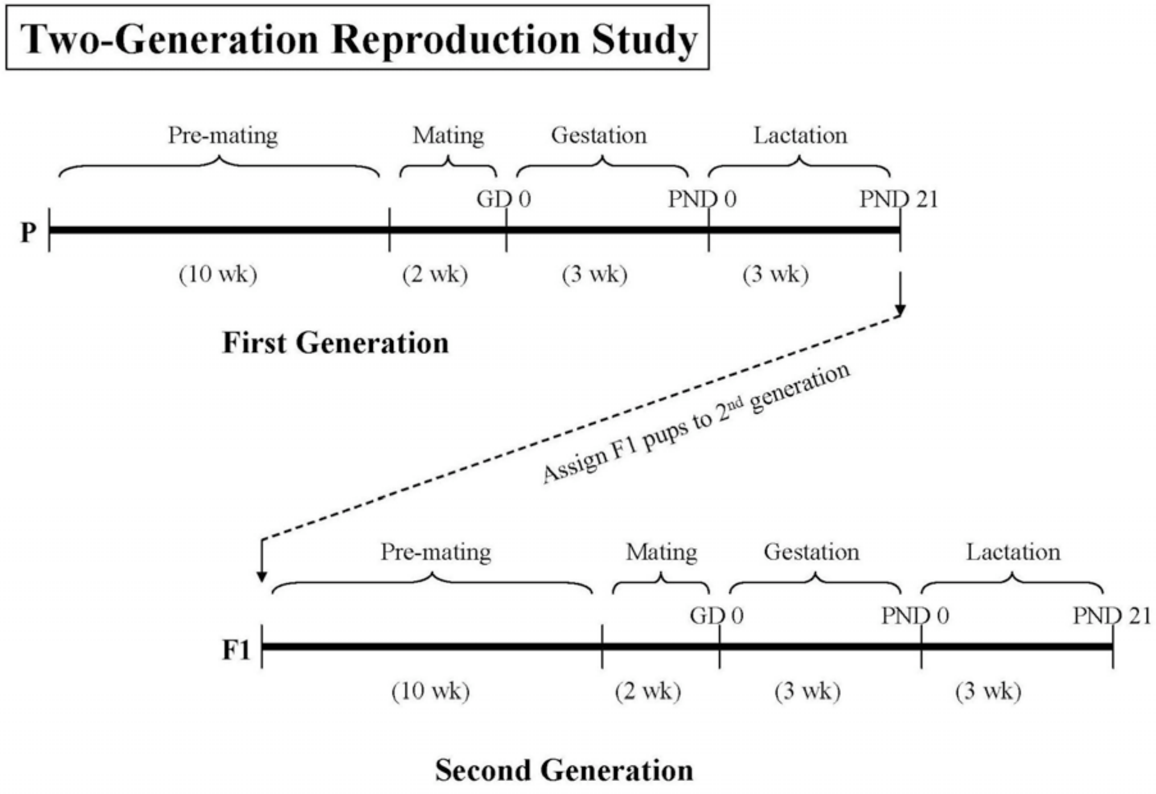
Two-Generation Reproduction Study
Pre-mating
Mating
Gestation
Lactation
GD 0
PND 0
PND 21
P
(10 wk)
(2 wk)
(3 wk)
(3 wk)
First Generation
Assign F1 pups to 2nd generation
Pre-mating
Mating
Gestation
Lactation
GD 0
PND 0
PND 21
F1
(10 wk)
(2 wk)
(3 wk)
(3 wk)
Second Generation

Developmental Neurotoxicity Study

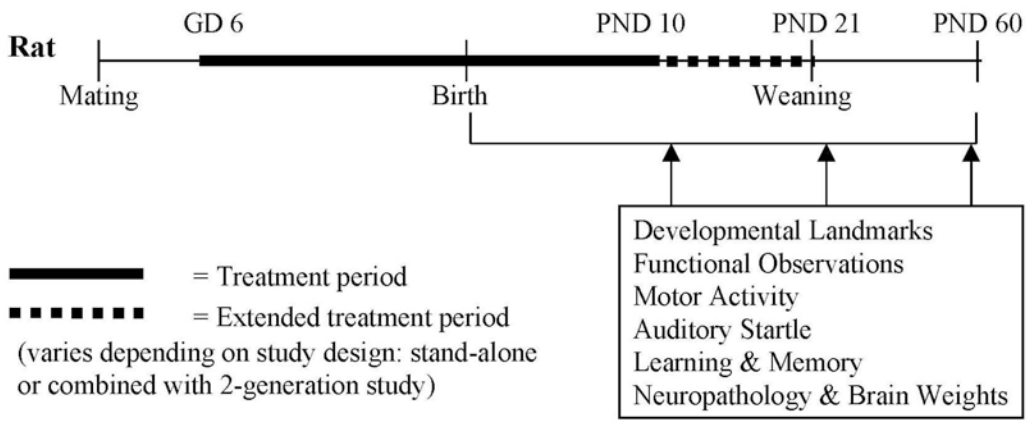

Extended One-Generation Reproduction Study

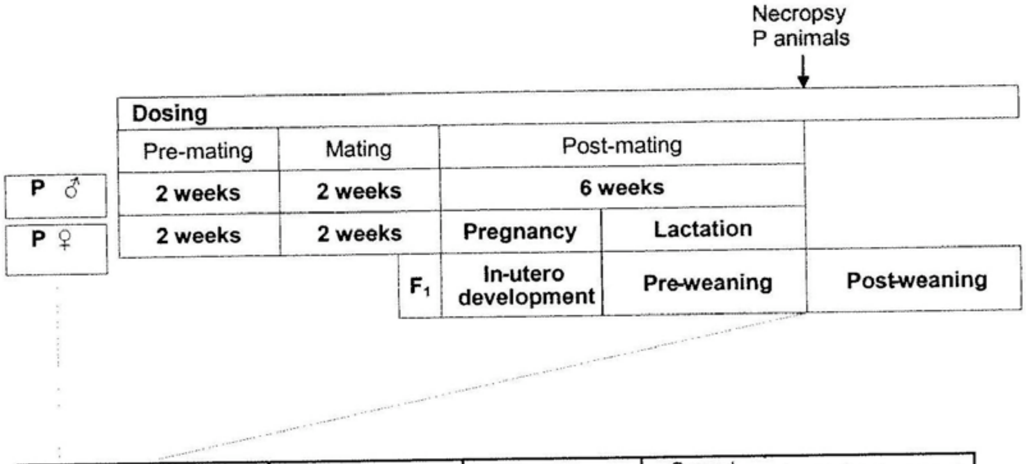

Parental generation	Cohort	Designation	Animals/Cohort	Sexual Maturation	Approximate age at necropsy (weeks)
Target is 20 litters per group	1A	Reproductive	20 M +20 F	Yes	13
	1B	Reproductive	20 M +20 F	Yes	14 or 20 if triggered
	2A	Neurotoxicity	10 M +10 F@	Yes	9
	2B	Neurotoxicity	10 M +10 F@	No	3
	3	Immunotoxicity	10 M +10 F@	Yes	8
	Surplus	Spares		No	3

@ one per litter and representative of 20 litters in total where possible

Figure 2. Mammalian *in vivo* endocrine assays
Timelines for study conduct are presented for the uterorophic assay (OECD 2007b, U.S. EPA 2009a), the Hershberger assay (OECD 2009a, U.S. EPA 2009b), and the male and femal pubertal assays (U.S. EPA 2009c,d). Duration of treatment is indicated in each diagram.

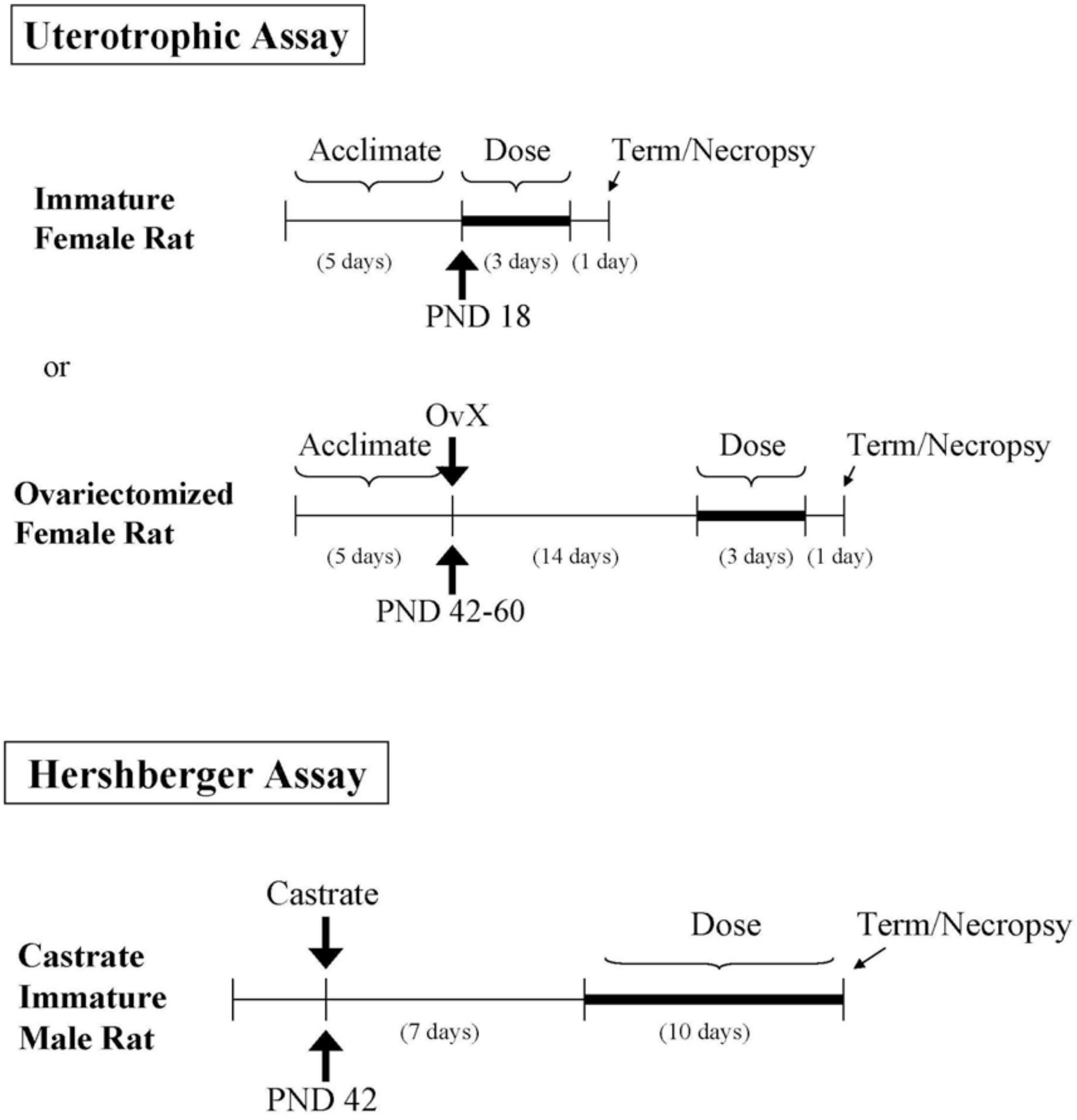

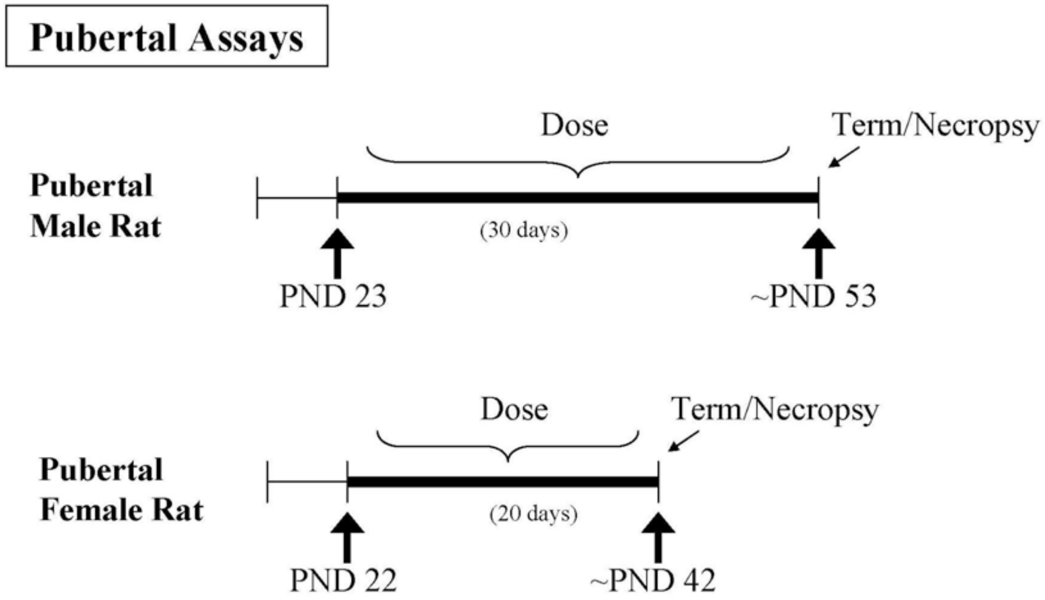
Pubertal Assays
Dose
Term/Necropsy
Pubertal Male Rat
(30 days)
PND 23
~PND 53
Dose
Term/Necropsy
Pubertal Female Rat
(20 days)
PND 22
~PND 42

Figure 3. Subacute, subchronic, and chronic/carcinogenicity studies in rodents
Timelines for study conduct are presented for subacute (OECD 2005, U.S. EPA 2000c), subchronic (OECD 1998, U.S. EPA 1998c), and chronic/carcinogenicity studies (OECD 2009b,c, U.S. EPA 1998d,e,f, 2001). Treatment is continuous from study initiation until termination. In the chronic/carcinogenicity study, dashed lines indicate a non-standard study phase: the perinatal exposure segment is unique to NTP studies, and the satellite group recovery period is considered optional.

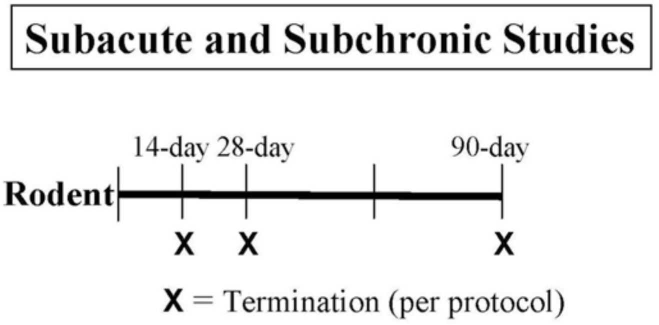

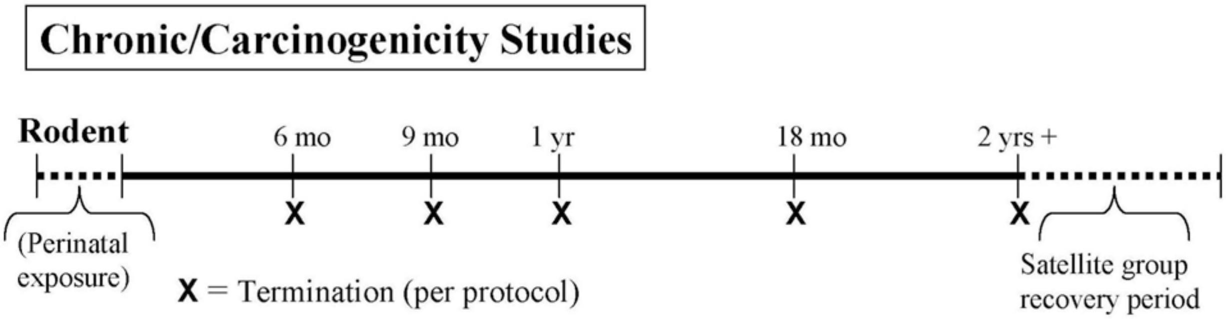

References:

Chapin R.E., Sloane R.A. 1996. Reproductive assessment by continuous breeding: evolving study design and summaries of ninety studies. Environ Health Perspect 105 (Suppl 1):199-395.

OECD. 1983. Test Guideline 415. OECD Guideline for Testing of Chemicals. One-generation reproduction toxicity study. Paris, France.

OECD. 1995. Test Guideline 421. OECD Guideline for Testing of Chemicals. Reproduction/ developmental toxicity screening test. Paris, France.

OECD. 1996. Test Guideline 422. OECD Guideline for Testing of Chemicals. Combined repeated dose toxicity study with the reproduction/developmental toxicity screening test. Paris, France.

OECD. 1998. Test Guideline 408. OECD Guideline for the Testing of Chemicals. Repeated Dose 90-Day Oral Toxicity Study in Rodents. Paris, France.

OECD. 2001. Test Guideline 416. OECD Guideline for Testing of Chemicals. Two-generation reproduction toxicity study. Paris, France.

OECD. 2005. Test Guideline 407. OECD Guideline for Testing of Chemicals. Repeated Dose 28-Day Oral Toxicity Study in Rodents. Paris, France.

OECD. 2007a. Test Guideline 426. OECD Guideline for Testing of Chemicals. Developmental Neurotoxicity Study. Paris, France.

OECD. 2007b. Test Guideline 440. OECD Guideline for Testing of Chemicals. Uterotrophic Bioasay in rodents: A short-tem screening test for oestrogenic properties. Paris, France.

OECD. 2009a. Test Guideline 441. OECD Guideline for Testing of Chemicals. Hershberger Bioassay in Rats: A Short-term Screening Assay for (Anti)Androgenic Properties. Paris, France.

OECD. 2009b. Test Guideline 452. OECD Guideline for the Testing of Chemicals. Chronic Toxicity Studies. Paris, France.

OECD. 2009c. Test Guideline 453. OECD Guideline for the Testing of Chemicals. Combined Chronic Toxicity/Carcinogenicity Studies. Paris, France.

OECD. 2010. OECD Guideline for the Testing of Chemicals. Draft Proposal for an Extended One-Generation Reproduction Toxicity Study. 17 November 2010. Paris, France. http://www.oecd.org/document/55/0,3343,en_2649_34377_2349687_1_1_1_1,00.html [accessed 24 November 2010]

U.S. EPA. 1998a. OPPTS 870.3800, Reproduction and Fertility Effects, Health Effects Test Guidelines, EPA 712-C-98-208, Washington, DC.

U.S. EPA. 1998b. OPPTS 870.6300, Developmental Neurotoxicity Study, Health Effects Test Guidelines, EPA 712-C-98-239, Washington, DC.

U.S. EPA. 1998c. OPPTS 870.3100, 90-Day Oral Toxicity in Rodents, EPA 712-C-98-199, Washington, DC.

U.S. EPA. 1998d. OPPTS 870.4100, Chronic Toxicity, EPA 712-C-98-210, Washington, DC.

U.S. EPA. 1998e. OPPTS 870.4200, Carcinogenicity, EPA 712-C-98-211, Washington, DC.

U.S. EPA. 1998f. OPPTS 870.4300, Combined Chronic Toxicity/Carcinogenicity, EPA 712-C-9-212, Washington, DC.

U.S. EPA. 2000a. OPPTS 870.3550, Reproduction/Developmental Toxicity Screening Test, Health Effects Test Guidelines, EPA 712-C-00-368, Washington, DC.

U.S. EPA. 2000b. OPPTS 870.3650, Combined Repeated Dose Toxicity Study with the Reproduction/Developmental Toxicity Screening Test, Health Effects Test Guidelines, EPA 712-C-98-207, Washington, DC.

U.S. EPA. 2000c. OPPTS 870.3050, Repeated Dose 28-Day Oral Toxicity Study in Rodents, Health Effects Test Guidelines, EPA 712-C-00-366, Washington, DC.

U.S. EPA. 2001. OPPTS 870.8355, Combined Chronic Toxicity/Carcinogenicity Testing of Respirable Fibrous Particles, EPA 712-C-01-352, Washington, DC.

U.S. EPA. 2009a. OPPTS 890.1600, Uterotrophic Assay, Endocrine Disruptor Screening Program Test Guidelines, EPA 740-C-09-010, Washington, DC.

U.S. EPA. 2009b. OPPTS 890.1400, Hershberger Bioassay, Endocrine Disruptor Screening Program Test Guidelines, EPA 740-C-09-008, Washington, DC.

U.S. EPA. 2009c. OPPTS 890.1500, Pubertal Development and Thyroid Function in Intact Juvenile/Peripubertal Male Rats, Endocrine Disruptor Screening Program Test Guidelines, EPA 740-C-09-012, Washington, DC.

U.S. EPA. 2009d. OPPTS 890.1450, Pubertal Development and Thyroid Function in Intact Juvenile/Peripubertal Female Rats, Endocrine Disruptor Screening Program Test Guidelines, EPA 740-C-09-009, Washington, DC.
